# Establishment and Validation of a Predictive Model in Female Patients with Obstructive Sleep Apnea

**DOI:** 10.1177/26884844251380142

**Published:** 2025-09-18

**Authors:** Wenxuan Yu, Shuwen Yang, Qinhan Wu, Shanqun Li, Huai Huang, Xiaodan Wu

**Affiliations:** ^1^ Department of Respiratory and Critical Care, Zhongshan Hospital affiliated to Fudan University, Shanghai, China.; ^2^ Department of Respiratory and Critical Care, Zhongshan Hospital, Fudan University (Xiamen Branch), Xiamen, China.

**Keywords:** obstructive sleep apnea, female, predictive model, nomogram

## Abstract

**Objective:**

To develop a noninvasive clinical diagnostic model based on clinical markers for obstructive sleep apnea (OSA) and to verify its predictive efficacy.

**Methods:**

A retrospective analysis was conducted on female patients who underwent diagnostic sleep monitoring and had complete medical records from January 2021 to April 2023 at Zhongshan Hospital affiliated with Fudan University. The risk factors were analyzed using LASSO regression and multivariate Logistic regression to construct a nomogram predictive model and evaluate its performance. Finally, the predictive efficacy of the constructed model was compared with that of the STOP-Bang score.

**Result:**

A total of 317 female patients were enrolled. Logistic regression analysis revealed that age (OR = 1.045, 95% CI: 1.02–1.072, *p* < 0.001), snoring (OR = 8.698, 95% CI: 3.439–24.89, *p* < 0.001), cerebrovascular disease (OR = 28.15, 95% CI: 2.408–931.7, *p* = 0.025), and Epworth Sleepiness Scale score (OR = 1.217, 95% CI: 1.112–1.348, *p* < 0.001) were independent risk factors for OSA in females, while insomnia (OR = 0.125, 95% CI: 0.03–0.423, *p* = 0.002) served as a protective factor. A nomogram predictive model was constructed using the aforementioned independent predictors, exhibiting good discrimination with a C-index of 0.881 (95% CI: 0.84–0.93) in the training cohort and 0.815 (95% CI: 0.73–0.90) in the validation cohort. Comparing the model’s area under the curve with that of the STOP-Bang score, the model’s predictive efficacy was found to be superior to the STOP-Bang score.

**Conclusions:**

The nomogram predictive model demonstrates good accuracy, consistency, and clinical utility. It aids doctors in the early identification of high-risk female patients with OSA in clinical practice, enabling timely preventive and interventional measures.

## Introduction

Obstructive sleep apnea (OSA) is a sleep-disordered breathing condition characterized by partial or complete upper airway obstruction, leading to decreased ventilation or even breathing pauses during sleep, causing chronic intermittent hypoxia and sleep fragmentation in patients.^1^

Currently, there is a rising trend in the prevalence of OSA among female populations, with recent studies have found that the overall prevalence of OSA in the adult population ranges from 9% to 38%, including 13% to 33% in men and 6% to 19% in women.^2^ Aside from typical symptoms like snoring, breathing pauses, daytime fatigue, and excessive daytime sleepiness, female patients often present with atypical symptoms such as insomnia, nocturia, morning headaches, mood disorders, cognitive decline, making diagnosis challenging.^3^ OSA patients commonly have comorbidities with other systemic diseases, and research indicates a significantly increased likelihood of cardiovascular events in women with OSA.^4^ Compared with men, female OSA patients are also more likely to have comorbid conditions like diabetes, thyroid disorders, asthma, and depression.^5^

The diagnosis of OSA relies on the polysomnography or home sleep testing (HST). Due to the costs, equipment, facilities, and expertise required for sleep monitoring, coupled with the prevailing notion that OSA primarily affects men, there is still insufficient understanding of female OSA, necessitating a better grasp of its clinical features and diagnostic levels. Several predictive models for OSA or severe OSA have been developed and demonstrate good predictive performance,^6^ yet these studies mostly focus on the general population or men, with limited research on the clinical features and risk factors of female OSA. This study aims to investigate the clinical characteristics of female OSA patients, explore their risk factors through logistic regression, and develop a prediction model to facilitate the identification of female OSA patients.

## Methods

This study focused on patients who underwent diagnostic sleep monitoring at Zhongshan Hospital, affiliated with Fudan University, from January 2021 to April 2023. Ethical approval for this study was obtained from the Medical Ethics Committee of Zhongshan Hospital, Fudan University.

Data collection included demographic information such as age, height, weight, body mass index (BMI), smoking history, alcohol consumption history, and symptom manifestations like morning headaches, daytime fatigue and sleepiness, snoring, gasping, breathing pauses at night, decreased memory and attention, as well as comorbid conditions including hypertension, diabetes, coronary heart disease, arrhythmia, hyperlipidemia, rhinitis, sinusitis, pharyngitis, along with scores from the Epworth Sleepiness Scale (ESS) questionnaire and the STOP-Bang questionnaire.

We included female patients with complete sleep monitoring data at our hospital and excluded those with comorbid psychiatric disorders, cognitive impairments, consciousness disorders, and patients under 18 or over 85 years of age.

### Epworth sleepiness scale

The scoring for evaluating the patient’s daytime sleepiness level consists of eight aspects. Scores are assigned as follows: “Never” = 0 points, “Rarely” = 1 point, “Sometimes” = 2 points, and “Frequently” = 3 points. The total score is then calculated. A higher score indicates a more severe level of daytime sleepiness.

### STOP-Bang questionnaire

The STOP-Bang risk assessment tool was developed by Canadian anesthesiologists and sleep experts in 2008 as a screening questionnaire. Initially designed for preoperative assessment, the questionnaire comprises 8 questions where each “Yes” response scores 1 point, and each “No” response scores 0 points. A total score of ≥3 points for the 8 questions indicates a high risk of OSA.

### HST

All participants refrained from napping, alcohol, strong tea, and coffee on the day of monitoring. They did not take sedative medications and underwent at least 7 hours of sleep monitoring overnight. The sleep monitoring was conducted using the American Cadwell Acumen7 portable sleep monitor. The CURATIVE SW-SM20000CB multi-channel sleep analysis diagnostic system was used to analyze sleep-related metrics, including the Apnea–Hypopnea Index (AHI), longest apnea duration, lowest oxygen saturation, average oxygen saturation, and Oxygen Desaturation Index (ODI).

Participants were divided into two groups: the OSA group and the non-OSA group based on the presence or absence of OSA. The diagnosis of OSA was guided by the 2017 “Clinical Practice Guideline for Diagnostic Testing for Adult Obstructive Sleep Apnea: An American Academy of Sleep Medicine Clinical Practice Guideline.”^7^

### Statistical analysis

Statistical analysis was performed by using the SPSS 27.0 software (SPSS Inc., Chicago, IL) and R Statistical Software (Foundation for Statistical Computing, Vienna, Austria). Categorical variables were presented as frequencies and percentages, and analyzed using the Chi-square test. The continuous variables were assessed for normality using the Shapiro–Wilk test. Data conforming to a normal distribution are presented as mean ± standard deviation (X¯ ± SD), while data with a non-normal distribution are presented as median and interquartile range. Inter-group comparisons were performed using the *t*-test or Mann–Whitney U test when appropriate. All statistical analyses were two-tailed tests, using a 95% confidence interval (CI). Results were considered statistically significant when *p* < 0.05. The LASSO regression was applied for variable selection. Multivariable logistic regression was performed to analyze the risk factors associated with OSA and to create a prediction model for identifying OSA from female. The nomogram was developed to predict the probability of OSA. The diagnostic performance was analyzed by the receiver operator characteristic curve (ROC) analysis.

## Results

In the sleep monitoring facility at Zhongshan Hospital, Fudan University, a total of 626 female participants who underwent sleep monitoring were extracted from records spanning from January 2021 to April 2023. Among these individuals, 293 had incomplete clinical or monitoring data, 13 did not meet the age requirements, and 3 had comorbid psychiatric disorders. Ultimately, 317 individuals were included in the study. They were randomly assigned to training and validation groups in a 7:3 ratio following standard research practices.

### Clinical characteristics

Among the 317 female participants, there were 186 individuals in the OSA group (AHI ≥5 events/h) and 131 individuals in the non-OSA group (AHI <5 events/h). A comparison of their demographics, symptoms, comorbidities, and questionnaire scores revealed that females in the OSA group were older and shorter in height compared with those in the non-OSA group. Snoring, nocturnal gasping and apnea, daytime fatigue, and excessive sleepiness were more prominent in the OSA group. In addition, they reported more perceived memory and attention impairments, while insomnia was less prevalent. Female patients in the OSA group were also more likely to have comorbidities such as hypertension, diabetes, heart disease, hyperlipidemia, and cerebrovascular disease. These differences were statistically significant (*p* < 0.05, Table 1). In addition, there were no significant differences between the training cohort and validation cohort. These results justified the use of the training and validation cohorts.

**Table 1.** Clinical Characteristics in the OSA and non-OSA Groups

	OSA group	non-OSA group	*p* value
Age, years	57 [46,66]	37 [30.5,50]	<0.001^*^
Height, cm	160 [157,164]	162 [158,166]	0.033^*^
Weight, kg	67 [60,77.9]	70 [60,80]	0.516
BMI, kg/m^2^	26.2 [23.7,29.7]	27 [23.1,29.9]	0.927
Smoking, *n* (%)	5 (2.69%)	8 (6.11%)	0.211
Alcohol, *n* (%)	12 (6.45%)	15 (11.5%)	0.172
Snoring, *n* (%)	167 (89.8%)	80 (61.1%)	<0.001^*^
Breathing pauses, *n* (%)	88 (47.3%)	26 (19.8%)	<0.001^*^
Daytime fatigue, *n* (%)	108 (58.1%)	53 (40.5%)	0.003^*^
Memory problems, *n* (%)	99 (53.2%)	32 (24.2%)	<0.001^*^
Morning headaches, *n* (%)	42 (22.6%)	23 (17.6%)	0.342
Insomnia, *n* (%)	5 (2.69%)	25 (19.1%)	<0.001^*^
ESS	6 [2,10]	1 [0,4]	<0.001^*^
STOP-Bang	4 [2.25,4]	2 [1,2.5]	<0.001^*^
Hypertension, *n* (%)	92 (49.5%)	24 (18.3%)	<0.001^*^
Diabetes, *n* (%)	23 (12.4%)	6 (4.58%)	0.030^*^
Heart disease, *n* (%)	35 (18.8%)	12 (9.16%)	0.026^*^
Hyperlipidemia, *n* (%)	69 (37.1%)	26 (19.8%)	0.001^*^
Upper respiratory diseases, *n* (%)	82 (44.1%)	73 (55.7%)	0.054
Cerebrovascular disease, *n* (%)	22 (11.8%)	3 (2.29%)	0.004^*^

^*^ Indicates a *p*-value of less than 0.05, representing a statistically significant difference.

Heart disease includes: coronary heart disease, arrhythmia; upper respiratory diseases include: rhinitis and sinusitis, pharyngitis.

### Nomogram for predicting OSA of female

**FIG. 1.**
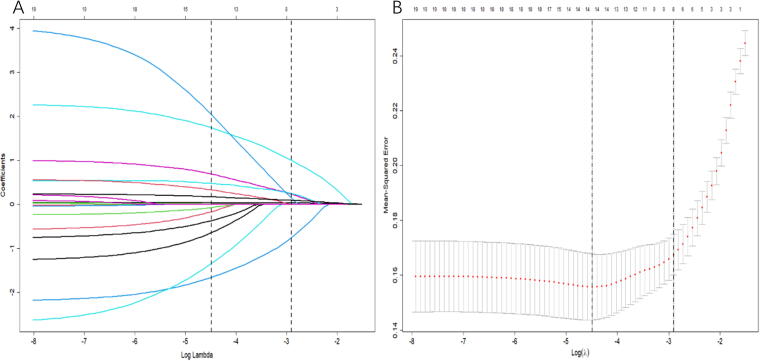
Features selection used the LASSO regression model in the features group. LASSO coefficients produced by the regression analysis **(A)**. λ = 0.0547 was chosen according to 10-fold cross-validation, where optimal resulted in 8 non-zero coefficients in the features group **(B)**.

As shown in Figure 1, we performed features selection using the LASSO regression model. Figure 1A represents the distribution of coefficients for each feature, where the coefficient profile was plotted against the log (λ) sequence. Figure 1B showed the adjustment of parameters in the LASSO model using 10-fold cross-validation to obtain a minimum standard. The binomial deviance was plotted versus log (λ). In Figure 1B, vertical dashed lines mark the optimal λ values: the leftmost line corresponds to the minimum deviance criterion, while the rightmost line represents the 1-standard-error rule (simplest model within one standard error of the minimum). Finally, we obtained 8 as the most significant features from the feature groups to construct the nomogram. These factors include age, snoring, nocturnal apnea, insomnia, hypertension, hyperlipidemia, cerebrovascular disease history, and ESS score.

**FIG. 2.**
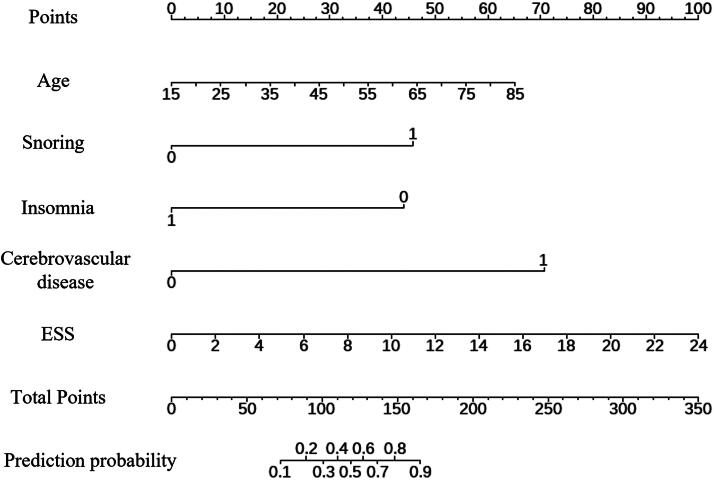
Nomograms were established to predict OSA for female patients. The nomogram will provide a graphical representation of the relative contribution of each factor to the overall risk of OSA in females. By calculating the individual score associated with each factor based on the nomogram, summing up these scores will lead to the total score, which corresponds to the predicted probability of females developing OSA.

Subsequently, these eight potential predictive factors were included in a multiple logistic regression model for analysis. The results indicated that age, snoring, history of cerebrovascular disease, and ESS score were independent risk factors for OSA in females, while insomnia was identified as a protective factor. To create a nomogram for individualized risk prediction of OSA in females using the five independent predictive factors mentioned earlier, each factor’s contribution to the risk prediction can be visually represented (Fig. 2). By incorporating age, snoring, history of cerebrovascular disease, insomnia, and ESS score into the nomogram, a personalized risk assessment tool can be developed.

### Validation of the nomogram

#### Discrimination ability

**FIG. 3.**
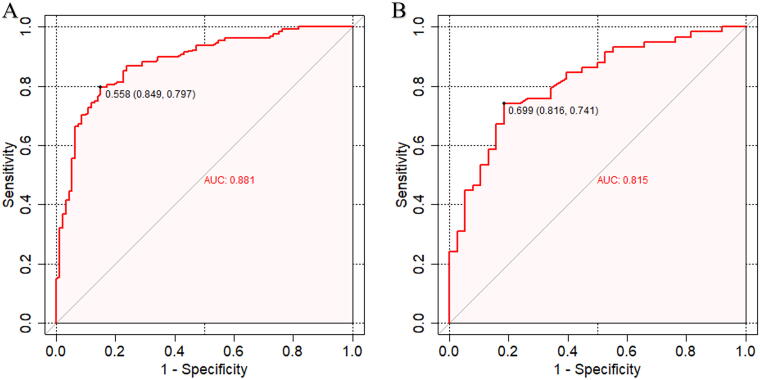
The x-axis is the “1-Specifificity,” and the y-axis is “Sensitivity.”

In both the training and validation cohorts, ROC curves were plotted (Fig. 3), indicating that the area under the curve (AUC) was 0.881 (95% CI: 0.84–0.93) for the training cohort (Figure 3A) and 0.815 (95% CI: 0.73–0.90) for the validation cohort (Figure 3B). These AUC values exceeding 0.8 suggest that the predictive model exhibits good accuracy in predicting OSA risk among females.

#### Calibration ability

**FIG. 4.**
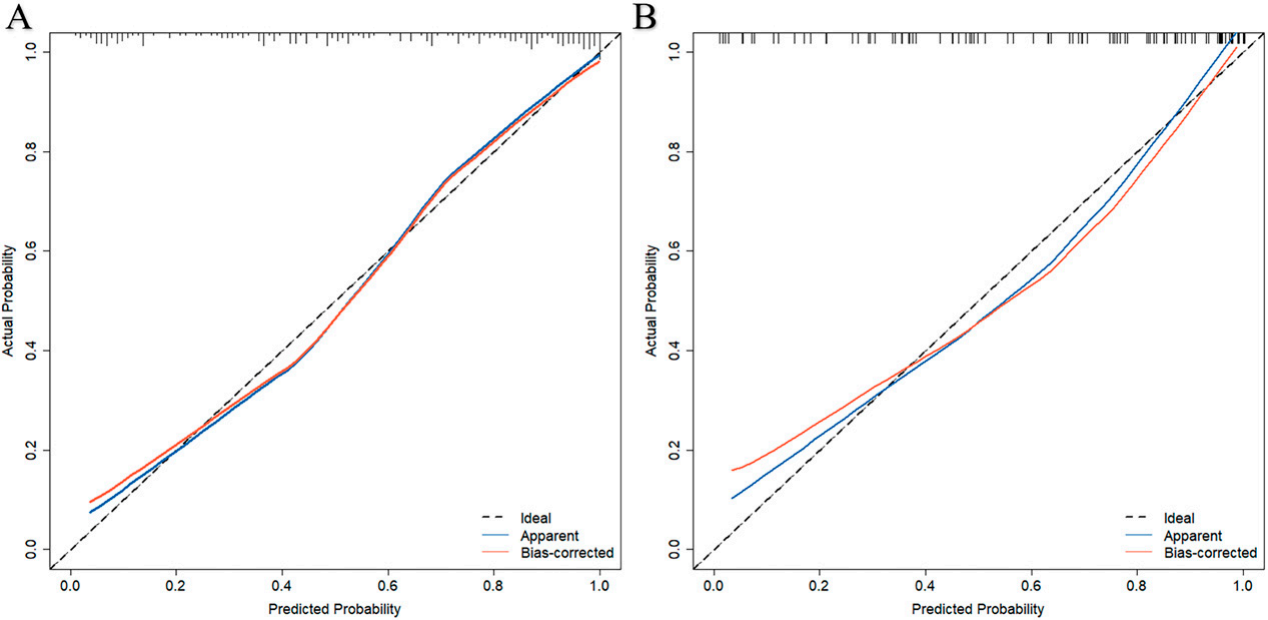
The ordinate represents the actual probability of OSA while the abscissa represents the nomogram-predicted probability of OSA. The diagonal dashed line means that the predicted probability is equal to the actual probability and the more deviation from the diagonal indicates the greater the error of prediction.

In both the training and validation cohorts, calibration curves were established (Fig. 4) with the x-axis representing the predicted probabilities of females having OSA and the y-axis showing the actual occurrence rates of OSA in females. The results revealed that the calibration curve aligned closely with the ideal fit line, indicating that the model exhibited good calibration. Furthermore, separate Hosmer–Lemeshow goodness-of-fit tests were conducted on the predictive model in the training and validation cohorts. The chi-square values were 8.67 and 10.67 respectively, with corresponding *p*-values of 0.47 and 0.30 (both >0.05). These results further confirm the good fit of the model, indicating that it adequately reflects the actual data in both the training and validation cohorts.

#### Calibration ability

**FIG. 5.**
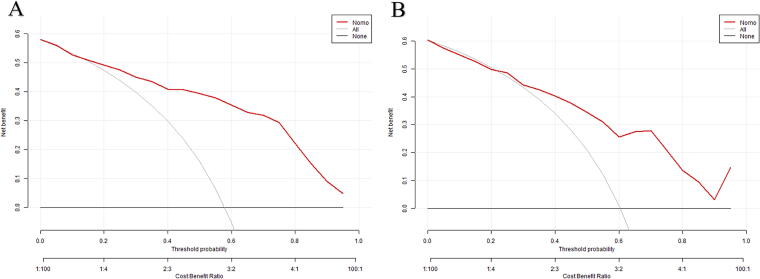
The abscissa shows the threshold probability, while the ordinate shows the net benefit. The gray line represents the assumption of all OSA positive cases, and the black line represents the assumption of all OSA negative cases.

Clinical decision curve analysis (DCA) curves were generated for both the training and validation cohorts (Fig. 5). The DCA plots depict the net benefit of the predictive model across a range of threshold probabilities, varying from 15% to 99% in the training cohort (Figure 5A) and 30% to 99% in the validation cohort (Figure 5B). It was observed that the net benefit curve for patients lies above the curves representing extreme scenarios (the gray horizontal line indicating no intervention for all patients and the gray parabolic line indicating intervention for all patients). This indicates that within these threshold probability ranges, the model exhibits clinical utility and has a higher net benefit for predicting OSA occurrence in females.

#### Performance comparison between the predictive model and STOP-Bang questionnaire

In a cohort of 317 female patients, the predictive performance of the proposed model was compared with the STOP-Bang score in this study. The ROC curve analysis revealed AUC values of 0.881 and 0.815 in the training and validation cohorts, respectively, outperforming the STOP-Bang score’s AUC of 0.797. Refer to Table 2 for a detailed comparison.

**Table 2.** Comparison Between the Predictive Model and STOP-Bang Questionnaire

	STOP-Bang	Training cohort	Validation cohort
AUC	0.797	0.881	0.815
Sensitivity	0.747	0.797	0.741
Specificity	0.748	0.849	0.816

## Discussion

Currently, there is a lack of a predictive model for OSA in females. This study examined the correlation between risk factors and severity of OSA in females by reviewing existing case data. It further explored the most significant independent risk factors and developed a visual nomogram as a user-friendly tool for predicting OSA in females, then validating its efficacy. The construction of the nomogram initially involved using LASSO regression to reduce 19 candidate variables to 8 potential predictive factors, including age, snoring, nighttime apnea, insomnia, hypertension, hyperlipidemia, cerebrovascular disease, and ESS score. These factors were then utilized in logistic regression analysis to build the predictive model and visualize the results. The model achieved a C-index of 0.881, and the calibration curve demonstrated close alignment between predicted and actual outcomes, indicating strong predictive performance. This study considered the characteristics of patients undergoing diagnostic sleep monitoring in outpatient settings, with readily accessible indicators that significantly enhance the identification of high-risk females with OSA in outpatient settings, aiding in disease diagnosis and management.

Recognized risk factors for OSA include obesity, age, gender, anatomical abnormalities in the upper airway, family history of OSA, long-term heavy alcohol consumption, sedative/hypnotic or muscle relaxant drug use, and long-term smoking, all of which can worsen OSA.^8^ In this retrospective study, 317 females undergoing diagnostic sleep monitoring in outpatient settings were surveyed. The analysis of various factors between the OSA and non-OSA groups revealed that OSA was more common in older females. In addition, females with OSA were more likely to have comorbidities such as hypertension, diabetes, hyperlipidemia, and coronary heart disease, consistent with findings in the majority of current research.

Regarding obesity and hyperlipidemia, our study revealed a higher prevalence of hyperlipidemia in the OSA group compared to the non-OSA group (*p* < 0.05). However, no statistically significant differences were observed in weight or BMI between the two groups (median BMI: 26.2 vs. 2 kg/m^2^, *p* = 0.927), which challenges the conventional perspective that obesity is an independent risk factor for OSA. This discrepancy may be attributed to several factors. Firstly, OSA patients often exhibit increased neck circumference and fat deposition in the neck region, unlike non-OSA individuals. BMI may not accurately reflect the distribution of fat within the body. In addition, the study population exclusively comprised women referred for diagnostic sleep monitoring due to clinical suspicion of OSA . This recruitment strategy might introduce selection bias, as referring clinicians may prioritize patients with overt symptoms or traditional risk factors (*e.g.,* obesity), while underrepresenting those with atypical presentations (*e.g.,* cognitive impairment, mood disorders) or subclinical OSA. To mitigate such bias, future studies should adopt standardized referral protocols aligned with guideline-based risk stratification and recruit cohorts from diverse settings (*e.g.,* primary care, population screenings) to enhance phenotypic heterogeneity.

Concerning clinical presentation and questionnaire assessment, this study observed that female individuals in the OSA group exhibited more prominent snoring, nighttime gasping and apnea, daytime fatigue, and excessive daytime sleepiness. Moreover, they reported more perceived memory and attention impairments, higher ESS scores, and STOP-Bang scores, consistent with clinical observations of OSA. Notably, the proportion of individuals experiencing insomnia in the OSA and non-OSA groups was 2.69% and 19.1%, respectively, with a statistically significant difference (*p* < 0.001). In subsequent multifactorial logistic regression analysis, insomnia continued to exhibit significant statistical differences (OR = 0.125, 95% CI: 0.03–0.423, *p* = 0.002). This could be due to poor nocturnal sleep quality in OSA patients, leading to compensatory oversleeping or excessive daytime sleepiness, which may mask symptoms of insomnia. Furthermore, insomnia patients often remain awake during the night, resulting in shortened sleep duration, reducing the manifestation of snoring, apnea, and hypopnea events, and lowering their naturally occurring sleep respiratory parameters. This may partly explain the higher prevalence of insomnia in patients with lower AHI in this study.

However, some studies indicate that OSA can coexist with insomnia, known as comorbid insomnia and sleep apnea (COMISA). Patients with COMISA may not demonstrate compensatory oversleeping or excessive daytime sleepiness typical in OSA. In severe cases, these patients might have AHI <5 events/h and relatively normal sleep monitoring parameters. For these individuals, airway narrowing or obstruction during sleep triggers apnea, leading to oxygen desaturation, increased carbon dioxide levels, stimulation of the respiratory center, sleep arousal, fragmented sleep, exacerbating or causing insomnia.^9^ The presence of insomnia may prompt some patients to prolong their time in bed and increase daytime rest, along with higher caffeine intake, as an attempt to alleviate daytime sleepiness and fatigue. Paradoxically, these actions may worsen nocturnal insomnia, creating a vicious cycle. Controversies persist regarding the interrelationship and causality between OSA and insomnia.^10^ Future studies are needed to delve into various subtypes and internal factors to elucidate the relationship between OSA and insomnia further.

In a multifactorial logistic regression analysis, a significant statistical difference was observed concerning the history of cerebrovascular disease (OR = 28.15, 95% CI: 2.408–931.7, *p* = 0.025). The results suggest that a history of cerebrovascular disease is an independent risk factor for predicting OSA occurrence in females. Research indicates that the impact of cerebrovascular disease on brain function and physiology may lead to new sleep disorders, including sleep apnea and insomnia.^11^ Bassetti et al.^12^ further explored the relationship between mortality rates and AHI levels in stroke patients. They categorized patients postischemic stroke based on AHI (low: AHI <10 events/h; high: AHI ≥ 30 events/h) and followed-up on mortality rates within 60 months after stroke. The study revealed a higher mortality rate in the high AHI group (22%) compared with the low AHI group (9%), with deceased patients exhibiting significantly higher AHI levels than survivors (25 ± 20.1 vs. 16.7 ± 15.4 events/h, *p* = 0.04). Previous studies have confirmed a significant association between AHI levels and all-cause mortality 1–3 years and even up to 10 years poststroke follow-up.^13–15^ Brown et al.^16^ investigated various sleep parameters in relation to recurrent ischemic stroke (median time post-ischemic stroke: 1.66 years). The results indicated a significant association between ODI and recurrent ischemic stroke (HR 1.34, 95% CI: 1.02–1.76, *p* = 0.04), while apnea index (AI), hypopnea index (HI), and recurrent ischemic stroke showed no significant correlation. On the other hand, OSA patients experiencing nocturnal apnea and arousals activate the sympathetic nervous system, leading to increased plasma levels of adrenaline and noradrenaline, elevated blood pressure and intracranial pressure, reduced cerebral perfusion, significantly enhanced platelet aggregation, and increased thrombotic risk, thereby further elevating the risk of stroke.^17^ These findings collectively indicate a significant association between stroke and the presence or severity of OSA in patients, highlighting the importance of prioritizing sleep screening in stroke patients. Increased understanding of the relationship between the two pathologies is essential for devising more effective strategies targeted at identified risk factors.

## Limitation

First, this study is a single-center research with a relatively limited sample size. Future studies could consider increasing the sample size by including populations from different regions and centers to reduce bias. Second, due to the inevitable selection bias in the inclusion of the population, the universality of our nomogram for women living in the community or asymptomatic people still needs further verification. Future research should focus on evaluating the performance of the model in non-screened population (such as primary medical institutions or community screening scenarios) in a prospective design to verify its effectiveness in identifying OSA in people without clinical suspicion. Third, the independent variables in this study mainly consist of patients’ basic information, clinical manifestations, and comorbidities. Future research should also include upper airway anatomical structures and relevant blood biochemical markers to further clarify their correlation with OSA. By incorporating these aspects into risk prediction and enhancing the predictive capability of the model, a more comprehensive understanding of OSA and its associated factors can be achieved.

## Conclusions

The logistic regression model constructed using five features—age, snoring symptoms, history of cerebrovascular disease, ESS score, and insomnia—exhibits good accuracy, consistency, and clinical utility. This model can aid health care professionals in early identification of high-risk females for OSA, enabling timely implementation of preventive and intervention measures in clinical practice.

## Data Availability

Data will be available on reasonable request.

## Abbreviations Used

AHI: apnea-hypopnea index
AUC: area under curve
BMI: Body Mass Index
CI: confidence interval
COMISA: co-morbid insomnia and sleep apnea
DCA: Decision Curve Analysis
ESS: Epworth Sleepiness Scale
HST: home sleep test
LASSO: Least Absolute Shrinkage and Selection Operator
ODI: oxygen desaturation index
OR: odds ratio
OSA: obstructive sleep apnea
PSG: polysomnography
ROC: receiver operating characteristic
